# Three dimensional culture of fresh and vitrified mouse pre-antral follicles in a hyaluronan-based hydrogel: a preliminary investigation of a novel biomaterial for in vitro follicle maturation

**DOI:** 10.1186/1477-7827-10-29

**Published:** 2012-06-13

**Authors:** Nina Desai, Faten Abdelhafez, Anthony Calabro, Tommaso Falcone

**Affiliations:** 1 Cleveland Clinic Fertility Center, Department of OB/GYN and Women’s Health Institute, Cleveland Clinic Foundation, Beachwood, OH, USA; 2 Department of Biomedical Engineering, Lerner Research Institute, Cleveland Clinic Foundation, Cleveland, OH, USA

**Keywords:** Follicle, Hydrogel, 3D culture, Oocyte maturation, Vitrification, Extracellular matrix, Ovary

## Abstract

**Background:**

Folliculogenesis within the ovary requires interaction between somatic cell components and the oocyte. Maintenance of 3-dimensional (3-D) architecture and granulosa-oocyte interaction may be critical for successful in vitro maturation of follicles. Testing of novel biomaterials for the 3-D culture of follicles may ultimately lead to a culture model that can support the longer in vitro culture intervals needed for in vitro maturation of human oocytes from ovarian tissue biopsies.

**Methods:**

A novel tyramine-based hyaluronan (HA) hydrogel was tested for its biocompatibility with ovarian follicles. The HA was prepared at concentrations from 2 to 5 mg/ml. HA hydrogel was also formulated and tested with matrix proteins (ECM). Enzymatically isolated pre-antral follicles from the ovaries of 10–12 day SJL pups were divided amongst control (CT) and HA treatments. The growth of both fresh and vitrified follicles was assessed after encapsulation in the hydrogel. The basal culture medium was MEM alpha supplemented with FSH, LH, ITS and 5% FBS. Maturation was triggered by addition of hCG and EGF after in vitro culture (IVC). Outcome parameters monitored were follicle morphology, survival after IVC, antrum formation, GVBD and MII formation. Differences between treatments were analyzed.

**Results:**

HA and ECM-HA encapsulated follicles looked healthy and maintained their 3-D architecture during IVC. In control cultures, the follicles flattened and granulosa:oocyte connections appeared fragile. Estradiol secretion per follicle was significantly higher by Day 12 in ECM-HA compared to HA or CT (4119, 703 and 1080 pg/ml, respectively). HA and ECM-HA cultured follicles had similar survival rates (62% and 54%, respectively), percent GV breakdown (96–97%), MII formation (47–48%) and oocyte diameters at the end of IVC. Control cultures differed significantly in percent GVBD (85%) and MII formation (67%) . Vitrified-warmed follicles encapsulated in HA had an oocyte maturation rate to MII of 54% as compared to 57% in non-embedded follicles.

**Conclusions:**

Initial testing of this new and unique HA-based hydrogel was quite promising. The ease of follicle encapsulation in HA, its optical transparency and ability to be molded combined with its support of follicle growth, estradiol secretion and resumption of meiosis make this HA-hydrogel particularly attractive as model for 3-D ovarian follicle culture.

## Background

Ovarian tissue extraction and cryopreservation before chemo or radiation therapy may offer cancer patients new hope for preserving their fertility potential. Ovarian follicle culture in vitro and the production of mature competent oocytes presents many challenges. A variety of culture systems have been applied to follicle culture in different animal models as well as the human. The ground breaking work of Eppig et al. in 1989 resulted in the first live offspring from in vitro matured follicles grown on collagen impregnated gels [1]. Other investigators have been able to successfully mature follicles after growth on membranes coated with extracellular matrix [2-5], in multi-wells [6] or in microdrops [6-9].

The 3-D architecture of the follicle and the complex interactions between somatic cell components and the oocyte that are necessary for cytoplasmic and nuclear maturation are difficult to maintain in these 2-D culture formats. Follicle flattening disrupts cell-to-cell communication. Granulosa cells attach to culture surfaces and break through the basal membrane as they proliferate. Data indicate that gap junctions between the oocyte and its surrounding granulosa cells are critical for sharing of paracrine factors and transport of specific amino acids to the oocyte [1,10,11]. Similarly, oocyte-derived secretions help to regulate granulosa cell proliferation and metabolic processes [12-14]. The length of time in vitro necessary for oocyte maturation varies with species. Human follicles may require as long as 85 days to mature [15]. This may be impossible to achieve in conventional culture systems.

Design of 3-D culture systems that mimic the in vivo environment of follicles have therefore been the focus of much research (reviewed [16-18]). This report describes the first application of a novel new tyramine-based hyaluronan (HA) hydrogel to the culture of enzymatically isolated mouse preantral follicles. We tested different encapsulation methods and HA gel concentrations to establish a successful culture model compatible with continued follicle growth. The ability of HA embedded follicles to reach specific benchmarks during in vitro maturation was examined. We were able to demonstrate that oocytes derived from HA embedded follicles could resume meiosis and produce mature metaphase II oocytes. This HA culture model was also tested with vitrified preantral follicles after warming.

## Methods

### Animals

C57BL6 female mice and SJl/J males were purchased from Charles River Laboratories, Wilmington, MA and maintained as a breeding colony. Animals were housed at the Cleveland Clinic’s Animal Care facility within an environment of controlled temperature and lighting (12 h dark:12 hr light). Animals were handled according to NIH guidelines and following IACUC protocols of the Cleveland Clinic. Mice were sacrificed by cervical dislocation. Ovaries were dissected from 10–12-day old female mice for follicle isolation. Each experimental run consisted of ovaries from 3–6 pups. Data from two independent experiments was pooled.

### Follicle isolation and culture

Ovaries were placed in an organ culture dish containing pre-warmed Leibovitz-15 medium (Invitrogen; Carlsbad, CA) with 0.1% collagenase (Type I; Worthington Biochemical, Lakewood, NJ). The dish was placed on a laminar flow bench top at 37°C for 90 minutes. At 30 minute intervals, ovaries were transferred to a new dish containing L-15 medium and gently pipetted using Eppendorf pipette tips of decreasing bore size to facilitate release of follicles from underlying stroma. Undigested ovarian tissue was returned to the collagenase dish for further enzymatic digestion. The ovarian digest was visualized under a dissecting microscope at 40X magnification, and isolated follicles were collected with a finely drawn glass micropipette. Follicles were washed three times in culture medium to remove any traces of L-15 and enzyme. Culture was performed in α-modified Minimum Essential Medium (α-MEM; Invitrogen) supplemented with 5% fetal bovine serum, 100 mIU/ml FSH,10 mIU/ml LH, ITS (10 μg/ml insulin, 5 μg/ml transferrin and 5 ng/ml selenium; Invitrogen). Secondary follicles (100–130 μm, oocyte <65 μm) with a centrally located oocyte, surrounded by 2 or more layers of granulosa cells and enclosed within an intact basement membrane were selected for experiments.

Isolated follicles were used for fresh experiments and also cryopreserved.

### Follicle vitrification and warming

The protocol for vitrification and warming of isolated pre-antral follicles has been previously described in detail [19]. The basal medium for all vitrification solutions was L-15 with 20% synthetic serum substitute (SSS; Irvine Scientific; Irvine, CA). Follicles were equilibrated for 5 min in 2 M ethylene glycol (EG; Sigma, St. Louis, MO) followed by a 30–60 second incubation in vitrification solution containing 6 M EG with 0.3 M raffinose (Sigma). All steps were performed at room temperature (22–25°C)

Vitrified follicles were warmed by immersion in L-15 medium containing 1.0 M sucrose. Follicles were held at room temperature for 10 minutes. Follicles were then quickly transferred to L-15 medium without sucrose and incubated at 37°C for another 10 minutes. The final wash step was in follicle culture medium (supplemented α-MEM) pre-equilibrated at 37°C with 6% CO_2_. Intact follicles were pooled in a culture dish containing fresh medium. Follicles were embedded in 3 mg/ml of HA in groups of 5–7 follicles per gel as described below. Growth of vitrified-warmed follicles (n = 74) after HA- embedding or in standard culture wells was tested.

### Follicle encapsulation

A novel tyramine-based hyaluronan (HA) hydrogel (provided by A. Calabro, Cleveland Clinic, Dept. of Biomedical Engineering) was tested for its biocompatibility with ovarian follicles. The HA was prepared at concentrations ranging from 2 to 5 mg/ml in either phosphate-buffered saline or Global medium (Life Global; Guilford, CT). HA was also formulated with extracellular matrix proteins (ECM) to try to further enhance follicle growth. Matrigel, an ECM preparation extracted from Engelbreth-Holm-Swarm mouse sarcoma (10 μg/ml; BD Biosciences; San Jose, CA) was added to HA in a ratio of 1:9. This was done on ice to prevent gelation of the ECM.

Aliquots of HA and ECM modified-HA (ECM-HA) were stored at −20°C and thawed just before use. The HA was activated for follicle embedding by addition of 5 μl of horse radish peroxidase (HRP) to 500 ul aliquots of HA. Cross-linking of HA was initiated by exposure of 25 μl of activated HA to one microliter of 0.03% hydrogen peroxide. Gels were rinsed in pre-equilibrated follicle culture medium before final plating.

We tested three different methods for embedding the pre-antral follicles in the HA: (1) Drops; (2); Microcapillary plugs; (3) Cylindrical beads. In Method 1, a one μl drop of 0.03% hydrogen peroxide and a 25 μl drop of activated HA were placed next to each other on a round glass coverslip. Follicles in groups of 7–10 were deposited into the HA drop, being careful not to track in too much medium. The HA drop with follicles was picked up with a microcapillary tube attached to a mouth pipet and then slowly expelled on top of the hydrogen peroxide. Gelation of the HA was generally completed within 3–4 minutes. The coverslip with HA gel drop was then placed in a 24-well dish with follicle culture medium pre-equilibrated at 37°C with 6% CO_2_ in air. In Method 2, the HA drop with 1–3 follicles was drawn up into a glass capillary tube, with the follicles in the middle of the HA fluid column. The tip of the capillary tube was then positioned over the hydrogen peroxide drop. A small amount of HA was expelled into the hydrogen peroxide, and then quickly drawn back in to the capillary tube to initiate gelling of the HA-follicle column. It was critical to avoid drawing air bubbles back up in to the capillary tube, since this interfered with gelling. The HA-gel plug with follicles was then gently expelled into a 24-well dish and pre-equilibrated medium was quickly added. Method 3 was similar, to the first method described, except that the HA was shaped into a bead as it gelled. This was done using two 21 G tuberculin syringes with the beveled needles bent at a 90 angle. Two minutes into the gelling process, the needles were used to repeatedly “push” the edges of HA drop until it formed a cylindrical bead with encapsulated follicles.

### In vitro follicle growth and oocyte meiotic competence

HA-gels containing follicles were placed individually in a 24-well plate with 800 μl of medium. Control wells were plated with a similar number of follicles per well. For each treatment, 6–18 wells were seeded, depending on total follicles available from the ovarian digest. After plating, the follicles were cultured at 37°C with 6% CO_2_ in air for 12 days. The medium was exchanged every other day by replacing one half of the culture volume with fresh equilibrated medium. During this in vitro culture (IVC) interval, follicles were imaged at each medium exchange.

At the end of the follicular growth phase, in vitro maturation was triggered by exposure of the follicles to culture medium supplemented with 1.5 IU/ml of human chorionic gonadotrophin (hCG), and 5 ng/ml of epidermal growth factor (EGF) (R&D Systems, Minneapolis, MN). Maturation was allowed to proceed overnight for 18–20 hours. Follicles were then exposed briefly to hyaluronidase (80 U/ml) to assist in removal from the gel. Encapsulated follicles were then dislodged from the HA gels using tuberculin syringes. Oocytes were denuded of granulosa cells by pipetting with finely drawn glass micropipets of decreasing diameter. After rinsing to remove residual enzyme, the oocytes were placed in 5 μl drops of fresh medium, separated according to initial well number and treatment group. Oocytes were assessed using an inverted microscope at 300X magnification. Each oocyte was photographed and its meiotic status documented. Live imaging of metaphase II oocytes for the presence of a meiotic spindle was performed using the Oosight Imaging system (CRI; Hopkinton, MA).

### Estradiol secretion

Levels of 17ß-estradiol were measured in conditioned media from encapsulated and control follicle cultures collected at various time points during IVC. For these measurements, conditioned medium was collected for each treatment at designated timepoints from three replicate wells containing 7–10 follicles. Estradiol was measured in duplicate using the Beckman Coulter Access 2 chemoluminescent immunoassay. Data was adjusted for number of follicles in each well by dividing the total measured estradiol secretion by the number of follicles at culture initiation. The sensitivity of the assay was 20 pg/ml.

### Follicle assessment and outcome parameters

Light microscopic evaluation of follicles was carried out using an Olympus IX-70 light microscope with Hoffman modulation contrast optics and imaging software. Culture wells were monitored for continued follicular growth and antrum formation. Images were captured for morphometric analysis on Day 1 (24 hours after plating) and at 2–3 day intervals until final maturation of oocytes to the metaphase II stage. Care was taken to image each of the individual follicles embedded in the gel. Follicle diameters were measured in duplicate from the outer edge of the thecal cell layer. Oocyte diameter was measured (excluding the zona) at the outset of the experiment and after the final maturation step. Additional measurements were taken during IVC if the oocyte could be visualized. Follicles in which the oocyte was no longer surrounded by granulosa cells or where the granulosa cells appeared dark and degenerative were classsified as non-viable. Calculation of antrum formation, GVBD and MII formation was based on viable follicles present at the end of the in vitro culture interval.

Outcome parameters used to evaluate the biocompatibility of the HA-hydrogel with ovarian tissue were estradiol secretion, follicle survival to the end of the in vitro culture interval, progressive increase in follicle size, ability to form an antrum, resumption of meiosis (GVBD) and oocyte maturation to the metaphase II stage.

### Statistical analysis

Statistical calculations were performed using Stats Direct software (Cheshire,UK). Statistical differences in follicle/oocyte diameters and estradiol secretion were analyzed using a two way ANOVA with repeated measures or a one-way ANOVA followed by the Tukey test for multiple comparisons. Categorical data was analyzed using the *χ*^2^ test for independent samples. Differences were considered to be significant if P values were <0.05.

## Results

### Biocompatibility and testing of HA concentrations

We wanted to first determine if HA was biocompatible with reproductive cells and the concentration range that might be most effective. The endpoint was ability to resume meiosis after 12 days of in vitro culture. HA was tested at four concentrations, 2, 3, 4 and 5 mg/ml.

In this experiment, Method 1 was used for embedding follicles. After 24 hours in culture, overt indications of cytotoxicity such as cellular degeneration and oocyte extrusion were absent. All four concentration of HA tested supported granulosa cell proliferation and in vitro follicular growth for 12 days. We observed that HA drops containing follicles had a tendency to flatten, especially at the lower HA concentrations. This affected follicle retention during IVC. Follicles located on the edges or sinking to the bottom of the gel, eventually ended up being extruded. Clearly, improvements to the encapsulation process will be needed to reduce follicle loss from the gel and assure maintenance of 3-D configuration in all embedded follicles.

**Figure 1:**
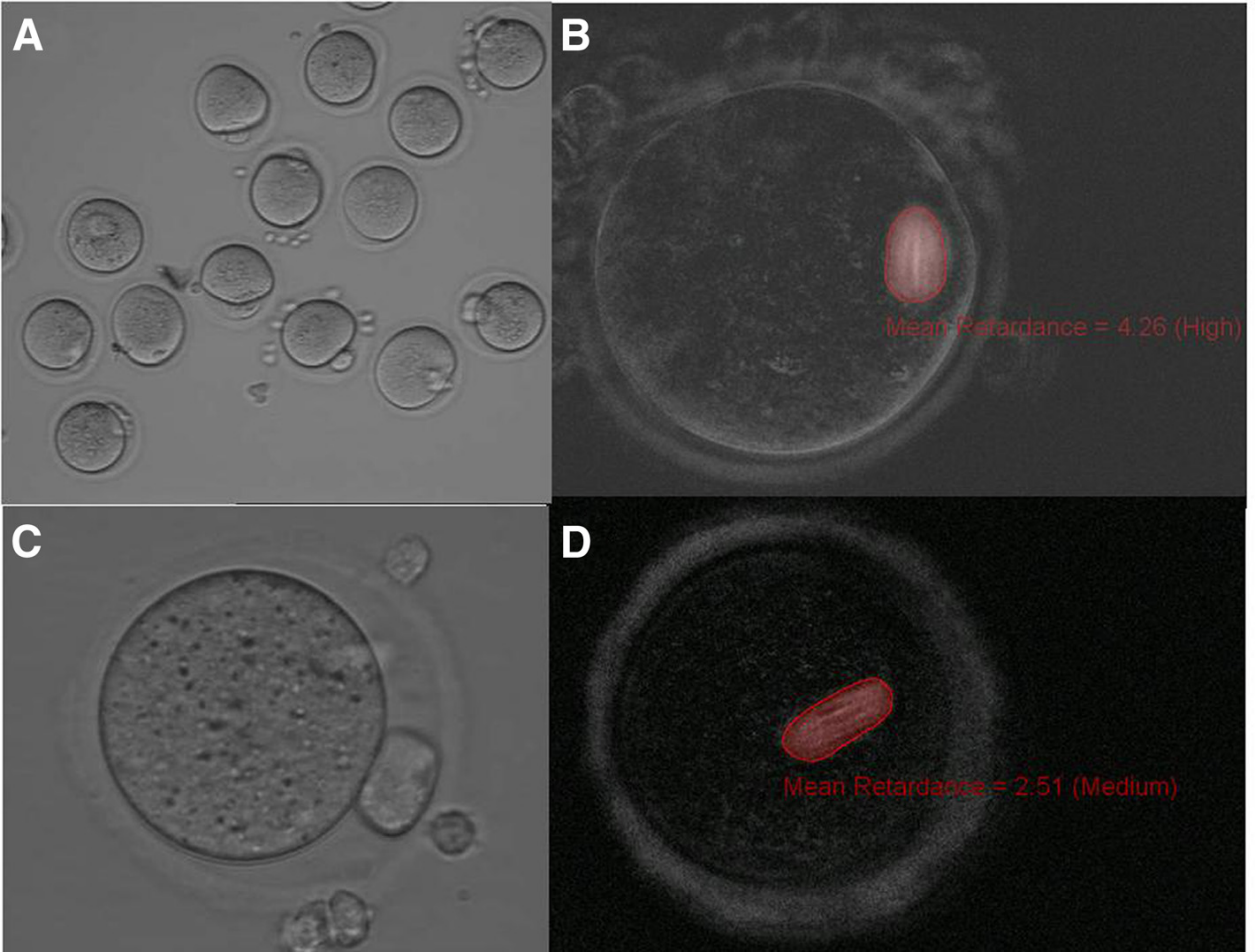
**Morphology of metaphase II oocytes and live imaging of meiotic spindles in oocytes derived from HA- embedded follicles (3 mg/ml), cultured for 12 days before exposure to hCG and EGF.** (A-D). Oocytes imaged at 300X magnification using the Oosight Imaging system . (C, D), Spindles showed moderate to high retardance, suggestive of a well organized and ordered structure.

Table 1 summarizes our results with the different HA concentrations tested. Amongst the follicles that remained embedded in the different HA treatments, 41–65% survived in vitro culture with an enclosed intact oocyte. Percent survival was not statistically different between the different HA gel concentrations. There was however a linear trend towards better survival with the lower gel concentrations (P = 0.039). The encapsulated follicles showed a continued increase in follicular diameter during the growth phase and retained their spherical shape. The resumption of meiosis after exposure to hCG and EGF indicate that HA was able to support follicular development in vitro. The proportion of oocytes capable of undergoing final maturation to metaphase II was not statistically different between HA concentrations. Polarized light imaging of metaphase II oocytes derived from HA gels revealed the presence of normal-looking meiotic spindles (Figure 1).

**Table 1:** HA gel concentration and follicle maturation

	**2 mg/ml**	**3 mg/ml**	**4 mg/ml**	**5 mg/ml**	**P-value**
Follicles initially plated	60	60	60	60	
Follicles retained in gel	34	48	36	44	
Survival * (%)	22 (65%)	24 (50%)	16 (44%)	18 (41%)	NS **
GVBD (%)	20 (91%)	24 (100%)	11 (69%)	15 (83%)	P < 0.05
MII (%)	12 (55%)	14 (58%)	7 (44%)	8 (44%)	NS

*Follicles with intact oocytes surviving 12 days in culture. Remaining follicles failed to develop and underwent atresia.

**Linear trend towards better survival with lower HA concentrations (P = 0.03). Follicles embedding in drop format.

### Testing of alternative follicle encapsulation methods

#### Microcapillary gel plugs

Based on the above results, we decided the 2 or 3 mg/ml HA might be best suited for follicle growth. We then explored alternate methods of follicle encapsulation to achieve better follicle retention within the hydrogel and prevent flattening. In the first experiment, follicles were embedded in a thin column of HA (10 ul) drawn into a microcapillary tube to shape the hydrogel (Method 2). The inner diameter of the microcapillary tube measured 1142 μm. We found that at the two extreme ends of the HA column, gelling was incomplete. Reducing the HA volume to 5 ul and the peroxide concentration to 0.015%, gave the most consistent results. The 2 mg/ml HA did not have enough rigidity for this format.

**Figure 2:**
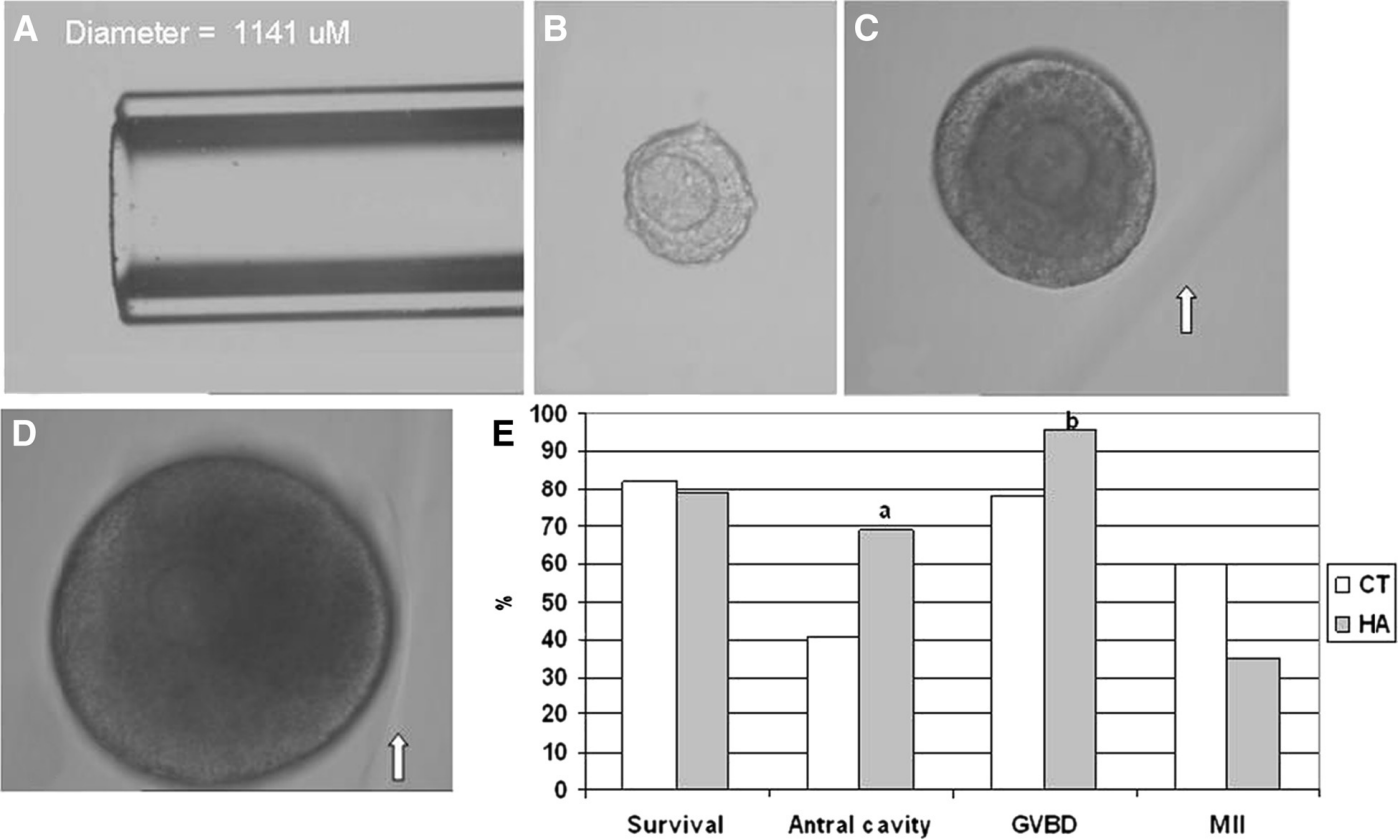
**Follicle encapsulation in microcapillary gel plug. A, Glass micopcapillary tube used for embedding.** Preantral follicle was mixed with HA (3 mg/ml) and drawn into a glass microcapillary tube. Cross-linking of HA was initiated and after 3–4 minutes the HA gel plug with follicle was expelled from the glass tube. Follicle growth was monitored; B, Preantral follicle on Day 3 of culture; C, Follicle with increasing diameter on Day 6 of culture. Edge of gel plug is indicated by arrow; D, Follicle on Day 10 of culture. Three dimensional architecture was preserved, and antral cavity formation was just visible. Follicle proximal to edge of gel plug (arrow) E, Pattern of follicle growth and oocyte maturation in conventional 2-D culture (CT) versus in the 3-D HA culture model. Antral cavity formation and GVBD was significantly higher in the HA gel (n = 33) as compared to CT (n = 71). Significant differences between groups are indicated by letters. a, P = 0.03; b, P = 0.007.

Figure 2 (A-D) illustrates a single follicle in a microcapillary plug. The 3-D architecture of follicles embedded in 3 mg/ml HA was well maintained through the culture interval. Antrum formation was clearly visible by Day 6–7 in growing follicles and oocytes resumed meiosis after hCG trigger. Figure 2E depicts the growth pattern of encapsulated follicles and controls plated in culture wells. Follicle viability was maintained in HA gels. No difference was noted in percent follicles surviving IVC. Antral cavity formation and oocyte progression to the GVBD stage stage was in fact significantly higher in the HA gels (69% and 96%, respectively) as compared to control cultures (41% and 78%, respectively). Despite these positive indicators and the resumption of meiosis, final oocyte maturation to metaphase II was lower after growth in the HA microcapillary gel plugs. The reduced HA volume and closer proximity of the cross-linking agent to the follicles during gelling within the HA column may have caused subtle damage.

Although the data was still encouraging, this method of embedding was not practical for handling large numbers of follicles. At best 1–3 follicles could be handled per microcapillary plug. Technically it was challenging to keep all the loaded follicles centered in the 5 ul HA column. If during cross-linking they shifted downwards, the potential of follicle damage as a result of inadvertent exposure to peroxide was high. Failure to gel was always an issue if mixing between the microliter of peroxide and the edge of the HA gel in the microcapillary tube was not adequate.

#### Cylindrical beads

The third method of follicle embedding that we explored involved a modification of the original drop technique to create a cylindrical bead with interspersed follicles (Method 3). By re-shaping the 25 μl HA drop in to a cylindrical bead, we hoped to create a more 3-D environment and lessen follicle extrusion. We once again found that the 3 mg/ml HA concentration was the easiest to work with. Follicles interspersed throughout the cylindrical bead could be readily visualized. All subsequent experiments were performed using this method of follicle encapsulation.

### Characterization of follicle growth in HA and ECM-modified HA

In this series of experiments, we characterized the growth pattern of secondary preantral follicles embedded in this novel tyramine-based hyaluronan (HA) hydrogel. We also looked at addition of ECM components to HA as a means to potentially improve follicle development and oocyte maturation. Control follicles were plated in a 24-well dish. Cylindrical beads containing 8–10 follicles were used and data was pooled from two independent runs. At the start of culture, the average diameter of follicles being distributed across the three treatment groups was 121.5 ± 12.9 μm. Average oocyte diameter was 57.2 ± 2.8 μm.

**Figure 3:**
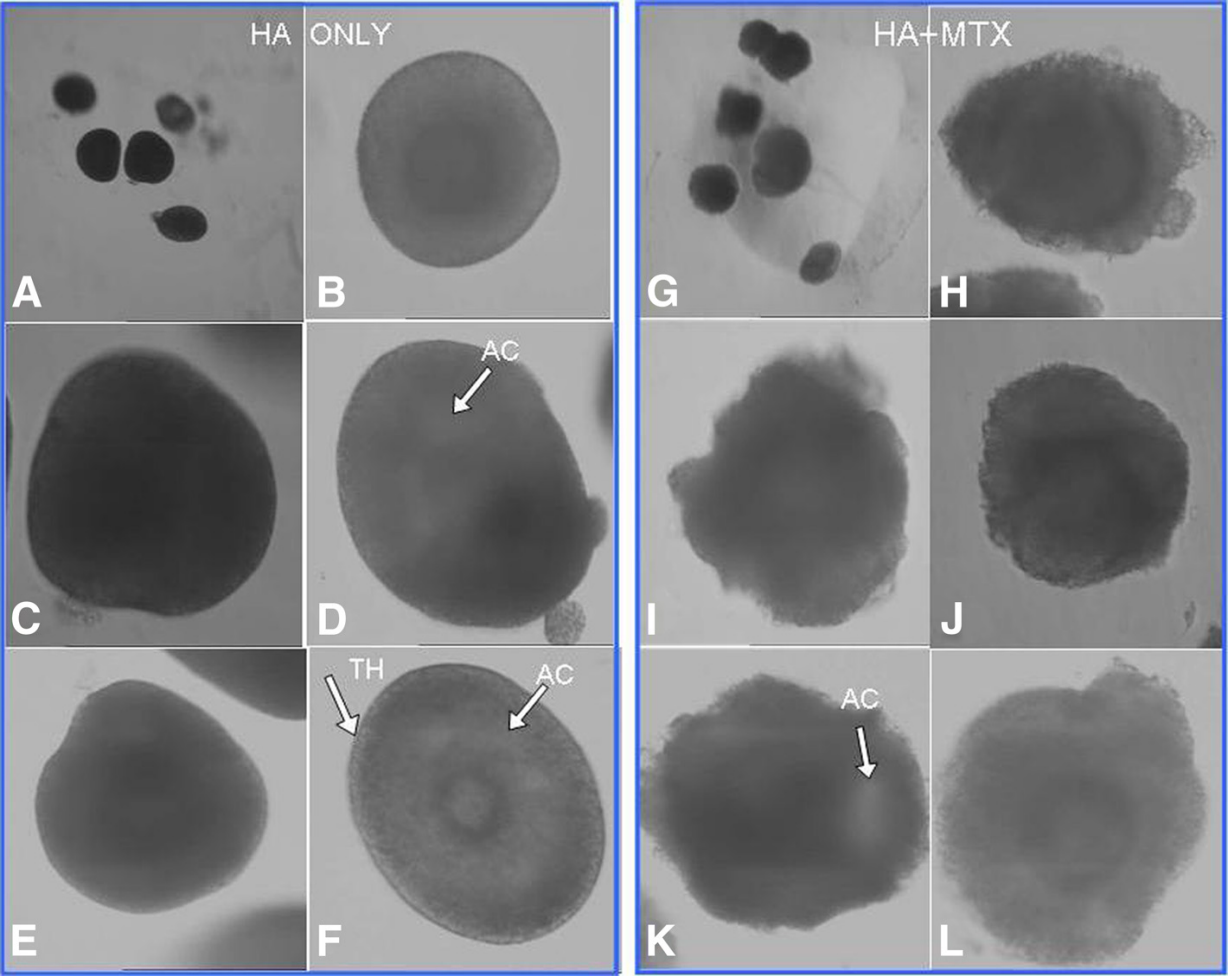
**Morphology of two layered preantral follicles embedded in 3 mg/ml HA gel or HA combined with extracellular matrix (ECM-HA) is depicted.** (A,G) Follicles from both culture models 24 hours after embedding. Follicle morphology on Day 7 in HA gel alone (B-D) or in ECM-HA (H-J). HA- embedded follicles grew in a more spherical fashion, with an intact basement membrane and thecal cell (TH) layer. Antral cavity (AC) formation is clearly visible (arrow). Follicle morphology in ECM-HA was quite different from HA alone but a 3-D growth pattern was maintained. By Day 12 of culture (E-F and K-L), follicles in both HA and ECM-HA gels formed antrums (arrows). Magnification 40X (Day 1), 200X (Days 7 and 12).

**Figure 4:**
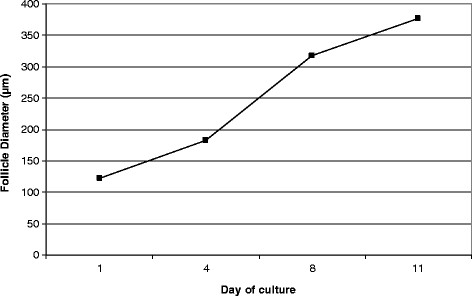
**The size of follicles encapsulated in HA beads (3 mg/ml) was monitored during the in vitro culture interval.** Data represent average diameter ± SD.

Typical morphology of preantral follicles and oocytes after 3-D culture in HA gels is shown in Figure 3. HA- embedded secondary follicles maintained their 3-D spheroid architecture throughout IVC. Multiple layers of granulosa cells formed around the oocyte. The diameter of HA-embedded follicles increased over the culture interval, measuring 385.6 ± 36.7 μm by Day 11 (Figure 4). Between Day 1 and 4, there was a 1.5 fold increase in diameter and mean follicle size was 183.4 ± 46.9 μm. Growth was accelerated from Day 4 through 11, resulting in a 2.1 fold increase in follicular diameter. An intact basal lamina with a thecal cell layer was typically observed.

In contrast, follicles in ECM-HA gels lost much of their spheroid symmetry by the fifth day of culture, as granulosa cells rapidly proliferated into the supporting matrix. A sharply delineated basal lamina and thecal cell layer was often difficult to see but follicular growth still continued in a 3-D fashion. Follicles in conventional 2-D culture flattened and lost their spherical shape within the first 3–5 days of culture. Granulosa cells proliferated and spread across the well surface. Oocytes became atretic and were spontaneously released from 25% of the follicles plated. By the 12th day of culture, granulosa:oocyte connections were tenuous, requiring very little to induce ovulation of the oocyte.

**Figure 5:**
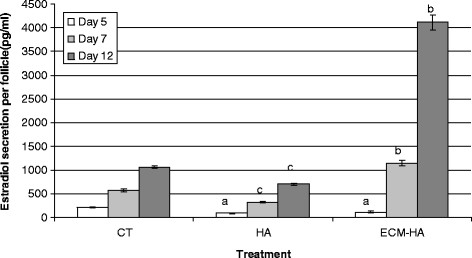
**Comparison of estradiol secretion in control versus HA and ECM- HA biomatrix.** Estradiol secretion by embedded follicles was significantly different from the control . On Day 5, level of estradiol was highest in conditioned medium from control follicles as compared to HA and ECM-HA (a, P < 0.001). No difference was found between HA and ECM-HA. By Day 7 and 12 of culture estradiol production by ECM- HA embedded follicles far exceeded that in HA alone or in the control CT (b, P < 0.001). At both of these time points, follicles in the HA treatment group secreted less estradiol than controls (c, P < 0.001).

The pattern of estradiol secretion by the preantral follicles in the three different culture systems was compared (Figure 5). There was progressive increase in estradiol secretion amongst the encapsulated follicles over time in culture, suggesting continued growth. HA and ECM-HA embedded follicles secreted half as much estradiol by Day 5 as control follicles (P < 0.001). Thereafter estradiol levels increased quite rapidly. HA follicle cultures showed an eight fold increase in estradiol levels by Day 12 (703.6 ± 19.5 pg/ml) as compared to the five-fold increase observed in controls (1060.5 ± 15.8 pg/ml). Follicles in the ECM-modified HA exhibited the highest secretion of estradiol (4119.1 ± 161.6; P < 0.001). This indicated a 37-fold increase in secretion of estradiol between Day 5 and Day 12.

**Figure 6:**
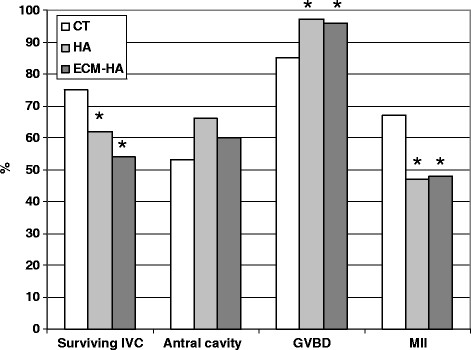
**Comparison of follicular growth in conventional culture wells (CT; n = 80) or in 3-D culture systems.** Follicles were embedded (groups of 8–10) in cylindrical beads of 3 mg/ml HA (n = 146) or ECM -modified HA (n = 166). *Significant difference between HA treatments and the control P < 0.05.

**Figure 7:**
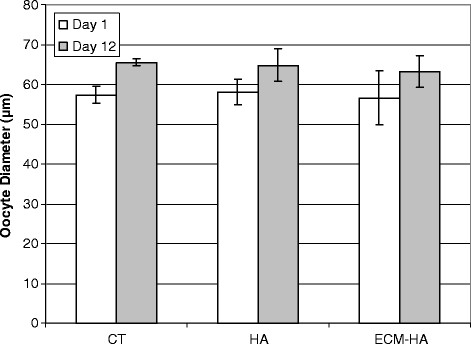
**Oocyte diameter excluding zona was measured on Day 1 (24 hours after plating) and on Day 12.** No significant differences were found between control and HA treatment groups.

The developmental performance of follicles embedded in HA or ECM-HA is shown in Figure 6. Antral cavity formation was similar between treatments. A significantly higher percentage of oocytes from HA and ECM-HA encapsulated follicles resumed meiosis and underwent GVBD when compared to the control. The transition from GVBD to the metaphase II stage was however somewhat lower in the HA treatments (HA 47%, ECM-HA 48%, CT 67%; P < 0.05). Final oocyte diameter after IVC was comparable in all three culture systems (Figure 7).

In a follow up experiment, we individually cultured and tracked HA-embedded follicles for antrum formation and correlated this to oocyte nuclear status after hCG/EGF trigger and meiotic spindle organization . The rate of antrum formation was 65% (26/40) . Oocytes derived from follicles with antrums completed meiosis and progressed to the MII stage (88%; 23/26). Spindles were observed with polarized light in 20 of the 23 MII oocytes examined.

### Development of cryopreserved follicles

Our last goal in this initial trial of HA embedding was to determine if the same methodology would support development of cryopreserved follicles. In this test run we assessed the developmental competence of cryopreserved follicles in this new 3-D culture system. Overall follicle survival after vitrification and warming was 92%. Warmed follicles were encapsulated in HA (n = 45) in groups of 5–7 and monitored for survival after IVC, GV breakdown and meiotic progression to the MII stage. This was contrasted to control wells with vitrified-warmed but non-embedded follicles (n = 29). No statistical difference was detected in any of the three parameters measured. Survival rate of HA-embedded follicles and the percentage of oocytes undergoing GVBD was 58% and 100%, respectively as compared to the control (79% and 96%, respectively). Oocytes derived from vitrified-warmed follicles embedded in HA were clearly able to mature to the metaphase II stage, much like control non-embedded follicles (54% vs 57%, respectively).

## Discussion

New evidence from 3-D culture of a variety of cell types have produced intriguing results, emphasizing the importance of a 3-D perspective when developing new culture systems [20]. This may be especially critical for follicle culture due to the complex nature of the interactions between the oocyte and the granulosa/thecal cells, the necessity of preserving communication through gap junctions and the length of time needed in culture for immature oocyte maturation. This report describes the first application of a new and unique hyaluronan- based hydrogel to the 3-D culture of ovarian follicles. Follicle architecture was maintained and diffusion through the gel during IVC was sufficient to support follicle growth, resumption of meiosis and oocyte nuclear and cytoplasmic maturation. Further modifications to follicle growth pattern were attempted by the addition of extracellular matrix components to the HA gel. This new model for 3-D in vitro follicle maturation was also successfully applied to vitrified mouse ovarian follicles after warming.

Encapsulation of ovarian follicles is one approach to maintaining the follicle’s 3-D architecture and preventing gap junction disruptions. This also allows sequestering of trophic factors in the vicinity of the growing oocyte. Both synthetic and biologic matrices have been used (reviewed [16,17]). The alginate model has been the most extensively studied 3-D system and has contributed much to our understanding of follicle cell biology [21-25]. Alginate seeded with pre-antral follicles can be formed into small beads, 500–1000 um in diameter by cross-linking in the presence of calcium. Alginate encapsulated follicles are responsive to FSH stimulation, secrete steroid hormones, express gap junction proteins, and yield mature oocytes capable of being fertilized and resulting in live offspring [21-23,25-31]. Despite accumulation of much positive data as regards follicle culture in alginate and the generation of morphologically normal looking oocytes, a recent study suggests that there may be some disturbance in meiotic spindle assembly [31]. The investigators suggest that this may contribute to lower developmental competence in oocytes derived from the alginate system. This is an interesting finding. It may well be time to also explore a totally different biomaterial for 3-D culture.

We have previously described the chemistry for the formation of tyramine -based HA hydrogels [32]. We and others are increasingly applying these hydrogels to a variety of tissue engineering and repair applications [32-35]. As a biomaterial, this HA hydrogel offers a new range of possibilities for studying follicle development in vitro. Hyaluronan is a naturally occurring glycosaminoglycan and a primary component of extracellular matrix, which makes it particularly attractive in designing 3-D scaffolding. By varying the HA concentration the rigidity of the hydrogel can be adjusted. The clarity of the hydrogel and its ability to be molded into a variety of shapes are other unique features of this gel that can potentially be exploited to create a physiologic environment for follicle growth.

The timeline of HA and ECM-HA embedded follicle growth mirrored that seen in the control. The basic benchmarks of in vitro follicle maturation such as increasing follicle diameter, estradiol secretion, antrum formation, increasing egg diameter and resumption of meiosis were all reached with this new culture system. The final oocyte diameter after in vitro maturation was equivalent for 2-D and 3-D cultured follicles, indicating that the HA matrix at the 3 mg/ml concentration allowed sufficient diffusion of nutrients to support continued growth.

Antrum formation in vivo may play a role in preventing hypoxia as the density of the granulosa cell layers surrounding the follicle increases [36,37]. The gene expression profile for follicles is different in culture environments that are permissive of antrum formation [25,38,39]. With 3D in vitro systems, adjustment of the rigidity of the matrix to the animal model may be vital for granulosa cell expansion and antrum formation [22,40]. It has been suggested that the more rigid gels lead to better outcomes in follicles from larger animal models [21,30]. Within the context of the present work the high rate of antrum formation at the 3 mg/ml concentration may be indicative of a ‘permissive” environment supportive of mouse follicle maturation.

Extracellular matrix (ECM) plays a vital role in regulation of folliculogenesis, influencing survival, granulosa cell proliferation, steroid production, cell behavior and structural organization [4,24,41-43]. The major components of ECM are collagen, laminin, fibronectin, proteoglycans and entactin. ECM proteins can affect in vitro maturation of follicles. Kreeger et al. reported increase in MII formation from 40% in alginate alone to 71% in alginate beads with either laminin or fibronectin [24]. We were hoping to see a similar improvement with the addition of ECM in the form of Matrigel to the HA. Granulosa cells in ECM-HA embedded follicles responded very well to the ECM proteins secreting far higher levels of estradiol and exhibiting rapid expansion of the granulosa cell layer as compared to follicles embedded in HA alone. This did not however translate into significantly higher formation of MII oocytes. The proportion of MII oocytes at the end of the culture interval was similar with both HA treatment groups. Matrigel is a very complex ECM product, containing not only basement membrane components but also a variety of associated growth factors such as IGF,TGF-ß, FGF and EGF. Perhaps the addition of specific ECM components to the HA gel will prove to be more beneficial. Creation of a more dynamic 3-D support matrix should also be explored. Shikanov et al. simulated such an environment by combining alginate with fibrin (FA-IPN) [25,38]. While the alginate could not be degraded, the fibrin was subject to proteolysis by secretions of the granulosa cells in the growing follicle. Theoretically, decreasing the rigidity of the biomatrix surrounding the follicle as it matures may better emulate the environment within the ovary itself.

HA and ECM-HA encapsulated follicles did not progress to MII at the rate observed in control cultures. This bears further scrutiny since both HA treatments had a significantly higher rate of GV breakdown. One possibility is that sequestration within the hydrogel may limit the process and degree of cumulus cell expansion. Cumulus cell expansion as a result of LH surge (or hCG exposure) has been shown to be critical for oocyte maturation and competence [44-47]. Antrum formation by follicles is closely linked to completion of meiosis. Further improvements of maturation rate may require removal of the follicles from the HA gel using hyaluronidase before the final incubation in maturation medium. Disturbance of granulosa:oocyte connections and denudation of the oocyte could however lead to spontaneous resumption of meiosis and GV breakdown. To obtain competent oocytes the process of nuclear as well as cytoplasmic maturation following hCG trigger need to coincide. With calcium alginate embedding, in the mouse but not other non-human primate systems follicles are removed from the hydrogel using alginase prior to the final maturation step [40,48,49].

We recognize that group culture of follicles has some limitations. It is possible that smaller non-growing follicles could exert a potentially negative influence on growing follicles and the overall maturation rate. The embedding technique needs further modification to efficiently handle large numbers of follicles cultured individually. Also for single follicle culture, embedding in smaller HA gels may prove necessary to improve diffusion towards the follicle. Multiple follicle growth in a single gel as we have done and the positive data gathered only emphasizes the potential of this matrix. Ultimately the creation of an “artificial ovary” will require the ability to seed numerous follicles in to a 3-D support structure.

The data presented raise many questions and further validation is certainly needed of this hyaluronan -based hydrogel system. The short in vitro culture interval needed for mouse follicle development makes it a valuable model for initial characterization and testing of biomatrices but has limitations. Although follicular architecture was maintained with 3-D growth in the hydrogel, this did not increase the rate of oocyte maturation. Gene profiling studies are underway that may help to identify differences between 2-D and 3-D mouse follicle growth. The true potential of 3-D vs 2-D culture systems may however become more obvious as we try to grow follicles from larger animal models as well as humans that require longer periods of in vitro culture. To date, effective technology for in vitro growth of human follicles and oocyte maturation has eluded us. Restoration of fertility through ovarian tissue grafting to orthotopic sites has been attempted with limited success. A biomatrix embedded with ovarian tissue fragments may be another avenue for tissue transplantation and needs further exploration.

## Conclusions

These data provide the framework for further testing of a new biomatrix for 3-D follicle culture. Bioengineering solutions are needed to make the embedding process more efficient and to control follicle position within the gel so as to get uniform diffusion of nutrients and gases in all directions and prevent follicle extrusion. Further optimization of the culture and hydrogel environment may be necessary to improve transition after GVBD to a mature functionally competent MII oocyte. Future studies need to focus on determining the functional competence of in vitro matured oocytes, their ability to be fertilized, form blastocysts and ultimately result in live offspring.

## Competing interests

ND,FA,TF had no competing interests. AC developed hydrogel and licensed product.

## Author contribution

ND,TF conceived this study. ND,FA developed the follicle culture system, designed protocols, carried out all experiments and analyzed the data. AC provided hydrogel and engineering expertise on biomaterial, properties and usage. ND drafted the manuscript. All authors read and approved the final manuscript.
